# Anomalous origin of the left circumflex artery from the pulmonary artery associated with non-compaction of the left ventricle: usefulness of multimodality imaging—a case report

**DOI:** 10.1093/ehjcr/ytad250

**Published:** 2023-05-25

**Authors:** Tom Bourcier, Serge Willoteaux, Alain Furber, Loïc Biere

**Affiliations:** Institut Mitovasc, UMR CNRS 6015-INSERMU1083, University of Angers, 3 rue Roger Amsler 49100 ANGERS, France; Department of Cardiology, University Hospital of Angers, 4 rue Larrey 49100 ANGERS, France; Institut Mitovasc, UMR CNRS 6015-INSERMU1083, University of Angers, 3 rue Roger Amsler 49100 ANGERS, France; Department of Radiology, University Hospital of Angers, Angers, 49000, France; Institut Mitovasc, UMR CNRS 6015-INSERMU1083, University of Angers, 3 rue Roger Amsler 49100 ANGERS, France; Department of Cardiology, University Hospital of Angers, 4 rue Larrey 49100 ANGERS, France; Institut Mitovasc, UMR CNRS 6015-INSERMU1083, University of Angers, 3 rue Roger Amsler 49100 ANGERS, France; Department of Cardiology, University Hospital of Angers, 4 rue Larrey 49100 ANGERS, France

**Keywords:** Case report, Coronary anomaly, Left ventricular non-compaction cardiomyopathy, Multimodality imaging, ACXAPA

## Abstract

**Background:**

The anomalous origin of the left circumflex artery from the pulmonary artery (ACXAPA) is a very rare coronary anomaly. Only a few cases have been reported until today, from incidental findings to autopsy reports after sudden cardiac death.

**Case summary:**

We report here for the first time the case of a man, previously monitored for asymptomatic left ventricular non-compaction cardiomyopathy, who presented with non-ST myocardial infarction and was diagnosed with ACXAPA. Complementary tests confirmed ischaemia in the corresponding territory, and the patient was referred to surgery for reimplantation of the circumflex artery.

**Discussion:**

Left ventricular non-compaction cardiomyopathy is a rare congenital cardiomyopathy whose association with coronary anomalies, not with ACXAPA, had previously been described until now. A related embryological origin could potentially explain this association. The management of a coronary anomaly should indicate dedicated multimodality cardiac imaging in order to not disregard the association with underlying cardiomyopathy.

**Learning points**

- The anomalous origin of the left circumflex artery from the pulmonary artery is a very rare coronary anomaly.
- Multimodality cardiovascular imaging should be mandatory following the finding of an anomalous origin of coronary arteries in order not to disregard underlying cardiomyopathy.
- Surgical strategies should be discussed in the light of coronary anatomical conformation and development of a collateral network.

## Introduction

Anomalous origins of coronary arteries occur in <2% of the general population. The true prevalence may be higher, as many subclinical cases never receive warrant medical attention.^1^ The left circumflex coronary artery arising either from the right sinus of Valsalva or directly from the right coronary artery is the most common coronary artery anomaly. Originating from the pulmonary artery is a rare finding^2^ and may affect the left coronary artery (ALCAPA), the left anterior descending artery (ALADAPA), or, infrequently, the left circumflex coronary artery [anomalous origin of the left circumflex artery from the pulmonary artery (ACXAPA)], as reported here.

The prevalence of ACXAPA is unknown, but a large review of 126 595 patients undergoing angiograms at a single site in the USA related just one case of ALADAPA (0.0008%) and no case of ACXAPA, confirming its rarity.^3^ Nonetheless, there are a few case reports available that depict a broad variety of presentation. Though some patients remain asymptomatic, it could be associated with severe clinical manifestations, ranging from acute heart failure, ventricular tachycardia, myocardial ischaemia, and sudden cardiac death.^4^

The association between congenital heart diseases, such as bicuspid aortic valve or aortic coarctation and ACXAPA, has previously been highlighted.^5^ This is the first case report of ACXAPA associated with left ventricular non-compaction cardiomyopathy (LVNC).

## Timeline

**Table:** 

Time	Event
**7 years prior to admission**	Diagnosis of left ventricular non-compaction cardiomyopathy.
**Up to admission**	Annual monitoring [echocardiography and Holter electrocardiogram (ECG)].
**Day 0 admission**	Non-ST elevation myocardial infarction–atrial fibrillation *de novo*, fast ventricular response (heart rate 150/min). Elevated troponin I at 2628 ng/L (normal < 40 ng/L).
**Day 1**	Spontaneous conversion to sinus rhythm. Troponin I peak at 4758 ng/L (normal < 40 ng/L). A coronary angiogram with suspicion of an anomalous origin of the left circumflex artery from the pulmonary artery (ACXAPA).
**Day 5**	ECG-triggered cardiac computed tomography. Confirmation of the diagnosis of ACXAPA.
**Day 8**	Stress single photon emission computed tomography showing ischaemia in three localized latero-basal segments.
**Day 14**	Discharge.
**Day 86**	Surgery: reimplantation of the circumflex artery into the aorta.
**Day 102**	Readmitted for recurrence of symptomatic atrial fibrillation. Amiodarone was added to nebivolol as a rhythm control strategy.

## Case

We report the case of a non-smoker, non-diabetic, and non-hypertensive 58-year-old man who had previously been monitored in our facility for LVNC with preserved ejection fraction.

**Figure 1:**
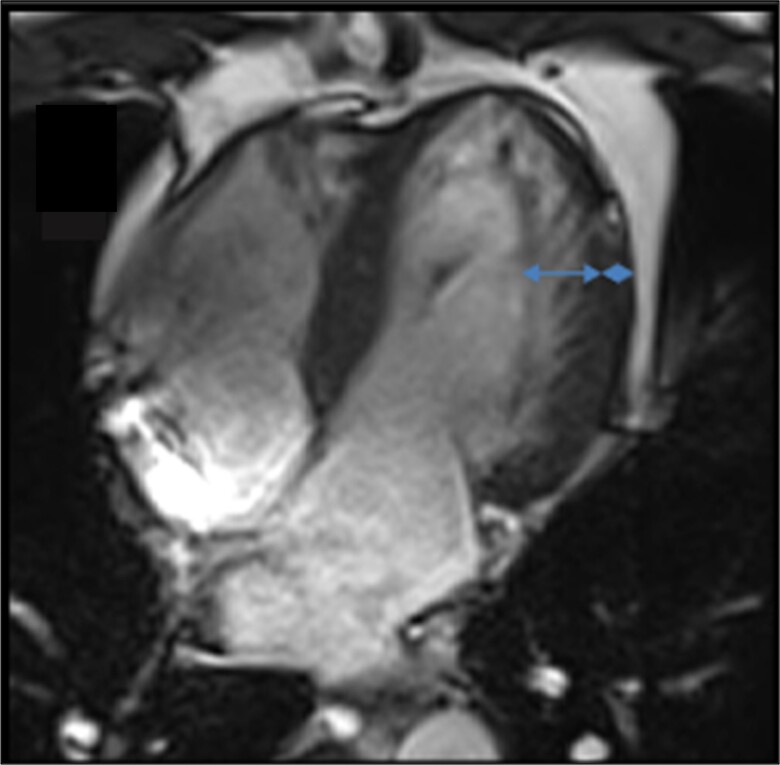
Cardiac magnetic resonance imaging, apical four-chamber view: calculated non-compacted/compacted myocardium ratio over 2.8 depicts left ventricular non-compaction cardiomyopathy involving apical and lateral walls.

The patient had been diagnosed with cardiomyopathy 7 years before this admission, after a routine echocardiogram for palpitation exploration and subsequent cardiac magnetic resonance (CMR) scan, to a diastolic non-compacted/compacted myocardium ratio > 2.3 (*Figure 1*). The initial functional stress test was negative. He declared no cardiac family history. Until this new admission, he had remained entirely asymptomatic, and he was being treated with nebivolol 2.5 mg once a day and followed up annually with echocardiography and Holter electrocardiogram (ECG).

**Figure 2:**
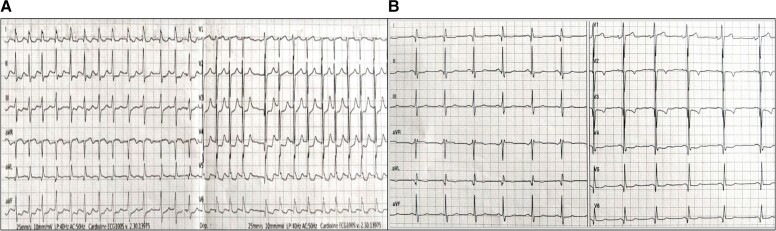
(*A*) Electrocardiogram at admission showing atrial fibrillation with a fast ventricular response (150 b.p.m.). (*B*) Electrocardiogram 6 h after admission showing sinus rhythm, pathological Q-waves in the lateral territory (leads V5-V6 and Lead I [DI], Lead augmented vector left [AVL]), and negative T-waves in the anterior leads (V2-V3).

He was newly referred to our cardiac emergency department for acute non-ST elevation myocardial infarction (NSTEMI) and atrial fibrillation *de novo* with a fast ventricular response. The initial clinical presentation included chest pain radiating to the left arm and palpitations. Clinical examination showed clear lung fields and heart sounds with an irregular pulse at 150 b.p.m. Blood pressure was measured at 106/89 mmHg, and he was afebrile. Initial workup confirmed atrial fibrillation with a fast ventricular response (*Figure 2A*) and elevated troponin of 2628 ng/L, which then rose to 4758 ng/L after 6 h (normal < 40 ng/L). The echocardiogram performed in the emergency room showed initially normal left ventricular ejection fraction at around 55 with evidence of lateral hypokinesia. Spontaneous conversion to sinus rhythm was attested few hours after the admission (*Figure 2B*).

In this context of NSTEMI with an intermediate GRACE risk score of 134, the patient was prescribed subcutaneous low-molecular-weight heparin (dosage: 0.5 mg/kg) combined with a loading dose of non-steroidal anti-inflammatory drugs (aspirin 250 mg intravenously) and a P2Y_12_ receptor inhibitor (ticagrelor 180 mg orally) before being referred for coronary angiography within 24 h.

**Figure 3:**
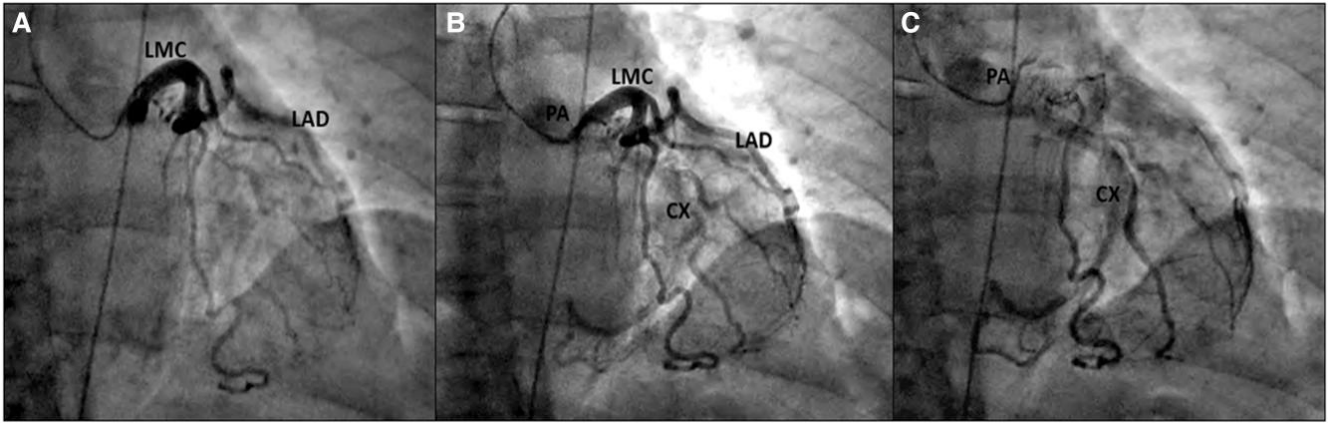
(*A*–*C*) Coronary angiogram right anterior oblique view shows retrograde filling of the circumflex artery, which is draining into the pulmonary artery.

**Figure 4:**
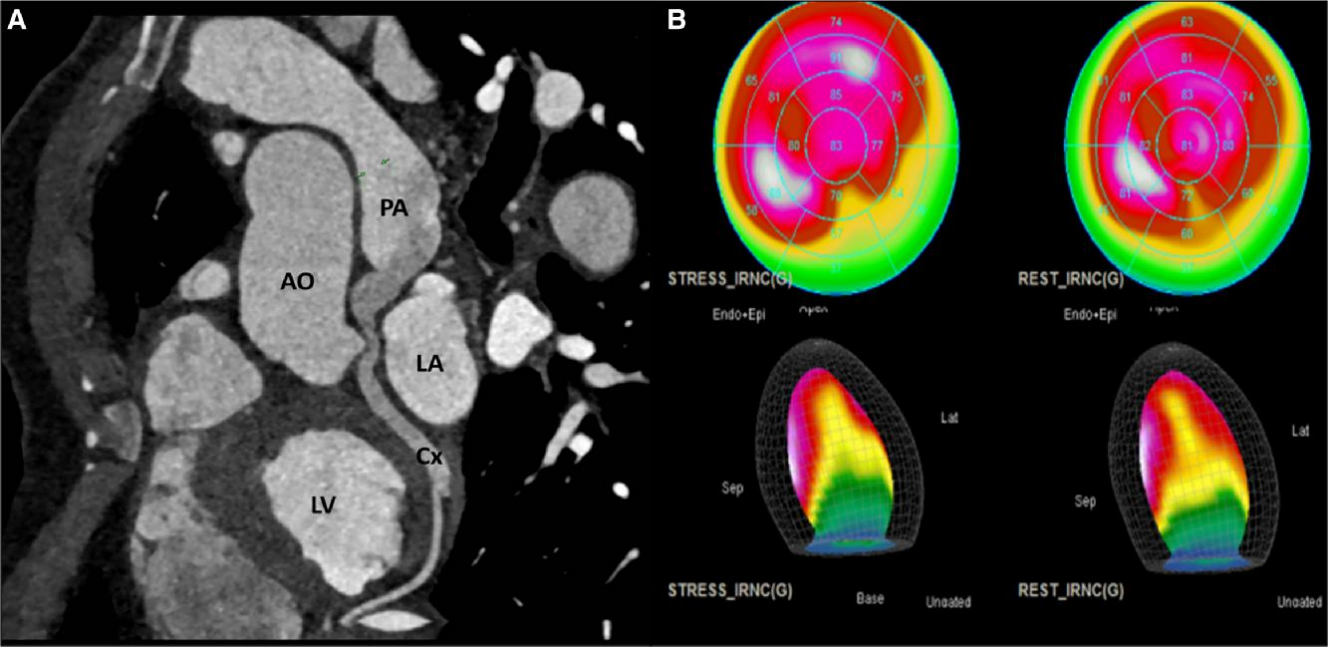
(*A*) Gated cardiac computed tomography: curved multiplanar reconstruction shows the abnormal origin of the circumflex artery from the pulmonary artery with aneurysmal deformation on the draining area. (*B*) Stress single photon emission computed tomography shows ischaemia in three latero-basal segments.

A coronary angiogram revealed abnormal retrograde filling of the left circumflex coronary artery via a collateral network vessel from the left anterior descending artery (*Figure 3A–C*). A consecutive gated cardiac computed tomography (CT) scan confirmed ACXAPA and the patency of the left circumflex artery with a steal phenomenon as it was not structurally occluded (*Figure 4A*). A stress myocardial perfusion single photon emission computed tomography (SPECT) showed localized latero-basal ischaemia (up to 2–3 segments/17) (*Figure 4B*) corresponding to the circumflex artery territory.

Knowing the evidence of ischaemia and the increased risk of sudden death, we referred the patient to surgery. After a multidisciplinary consultation, the preferred option was to reimplant the circumflex artery into the aorta (sinus) to prevent any residual coronary steal from the circumflex artery into the pulmonary artery, favoured by a pressure gradient, and surgical atrial fibrillation ablation was also performed.

He was successfully operated on 3 months later with an uncomplicated post-operative course.

He was readmitted to the hospital 16 days after surgery for a recurrence of atrial fibrillation. Amiodarone was added to nebivolol as daily therapy. Due to his CHAD2 VA2SC 1 and HAS BLED 0 scores, he was maintained on long-term oral anticoagulant treatment.^6^ He remained asymptomatic at the last follow-up.

## Discussion

We report here a case of LVNC combined with symptomatic ACXAPA, requiring cardiac surgery for reimplantation of the circumflex coronary artery.

The ‘coronary steal’ resulting from the abnormal connection into the pulmonary artery may have remained silent for several years thanks to the developed collateral network from the left anterior descending (LAD) artery, allowing efficient retrograde perfusion.^7^ Moreover, the relatively small area of the myocardium supplied by the LCX artery was probably another factor explaining the persisting asymptomatic state. The onset of atrial fibrillation *de novo* with a fast ventricular response may have triggered a sudden increased demand for oxygen of the non-compacted myocardium, leading to ischaemia, symptoms, and NSTEMI. Of note is that atrial fibrillation has been described as an independent predictor of mortality in LVNC patients.^8^

The link between ACXAPA and congenital heart disease was reported by Guenther *et al.*^5^, with a systematic review of 97 cases of ACXAPA or ALADAPA. Among these patients, 39 (40%) presented an association with congenital cardiac findings, such as bicuspid aortic valve or aortic coarctation mainly. Few cases described the association of dilated or localized hypertrophic cardiomyopathy combined with anomalous coronary arteries, suggesting a common pathogenic pathway.^9^

Left ventricular non-compaction cardiomyopathy prevalence remains unclear but may be as high as 9% in paediatric patients with congenital cardiomyopathy.^10^ Left ventricular non-compaction cardiomyopathy is the result of an arrest in the compaction process during the second month of embryological development. Its association with coronary artery abnormalities has already been described, with the hypothesis that the anomaly originated between the fifth and eighth weeks of intrauterine life when compaction and vascular development occurred.^11^ Coronary development involves progenitor cell migration to form a primitive vascular plexus, which attaches to the aorta allowing blood flow. The coronary arteries undergo remodelling, which ultimately leads to mature coronary vessels. During this period, the myocardium is meant to become thicker, making oxygen or nutrient delivery by simple diffusion impossible.^12^ We could raise the hypothesis that abnormal development of a coronary network during this critical period might be involved with the incidence of the non-compacted myocardium.

Because of the risk of heart failure, myocardial ischaemia, ventricular tachycardia, and sudden death, surgical intervention for the management of ALCAPA is recommended by ESC guidelines as Class I Level C.^13^ Bypass graft from the aorta for ACXAPA repair has also been considered but has been linked with long-term residual myocardial ischaemia even when the graft remains patent.^14^ Moreover, the extent of the collateral arterial network—resulting from the late diagnosis—may have made grafting more difficult to perform or made them non-functional by retrograde coronary blood flow. Therefore, we adopted a second option for this young patient, directly reimplanting the circumflex coronary artery into the aorta, which has always been assessed to be safe.^15^

Therefore, management of dedicated multimodality cardiac imaging should be required in order not to overlook the association of anomalous coronary arteries with cardiomyopathy. The diagnosis of LVNC should imply coronary assessment, *per se*.

## Conclusion

We report here the first case to associate ACXAPA and LVNC. A comprehensive and multimodal assessment of both the myocardium and coronary arteries should be considered when diagnosing either of these congenital conditions.

## Supplementary Material

ytad250_Supplementary_Data

## Data Availability

The data underlying this article are available in the article and in its online Supplementary material.

## Lead author biography

Tom Bourcier is a cardiology resident based in Angers, France. He has a master’s degree in basic cardiovascular research and is training in interventional cardiology.

## Supplementary material

**Slide sets:** A fully edited slide set detailing this case and suitable for local presentation is available online as Supplementary data.

**Consent:** The authors confirm that written consent for submission and publication of this case report including images and associated text has been obtained from the patient in accordance with COPE guidelines.

**Funding:** None declared.
