# Supplementary material for: Anomalous origin of the left circumflex artery from the pulmonary artery associated with non-compaction of the left ventricle: usefulness of multimodality imaging—a case report
Source: Eur Heart J Case Rep. 2023 May 25;7(6):ytad250. doi: 10.1093/ehjcr/ytad250 (PMC10265959; doi:10.1093/ehjcr/ytad250)
Supplement: ytad250_Supplementary_Data [file ytad250_supplementary_data.zip › Annexe figure.docx]

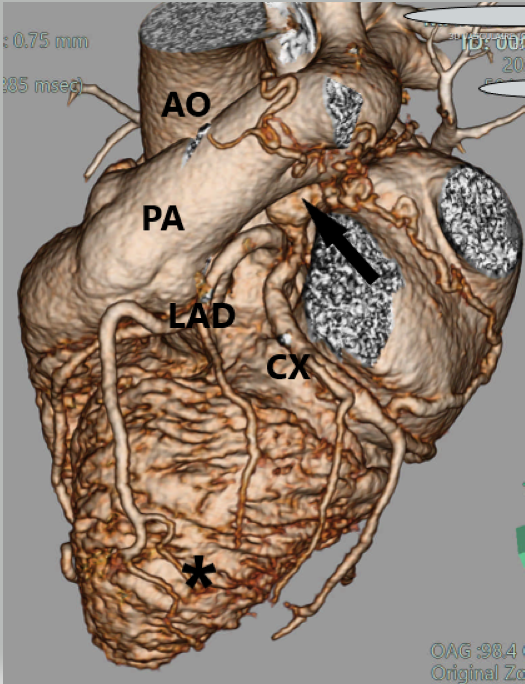


**Annexe** Synchronized cardiac computed tomography with volume rendering technic reconstruction showing aneurysmal deformation of the draining area of CX artery into PA (**arrow**) and developed collateral network linking CX with LAD (*****). **AO : Aorta, PA : Pulmonary artery, LAD : left anterior descending artery Cx : Circumflex artery,**
