# Supplementary material for: Anomalous origin of the left circumflex artery from the pulmonary artery associated with non-compaction of the left ventricle: usefulness of multimodality imaging—a case report
Source: Eur Heart J Case Rep. 2023 May 25;7(6):ytad250. doi: 10.1093/ehjcr/ytad250 (PMC10265959; doi:10.1093/ehjcr/ytad250)
Supplement: ytad250_Supplementary_Data [file ytad250_supplementary_data.zip › EHJ-CR-Slide-Set [Enregistrement automatique].pptx]

## Slide 1
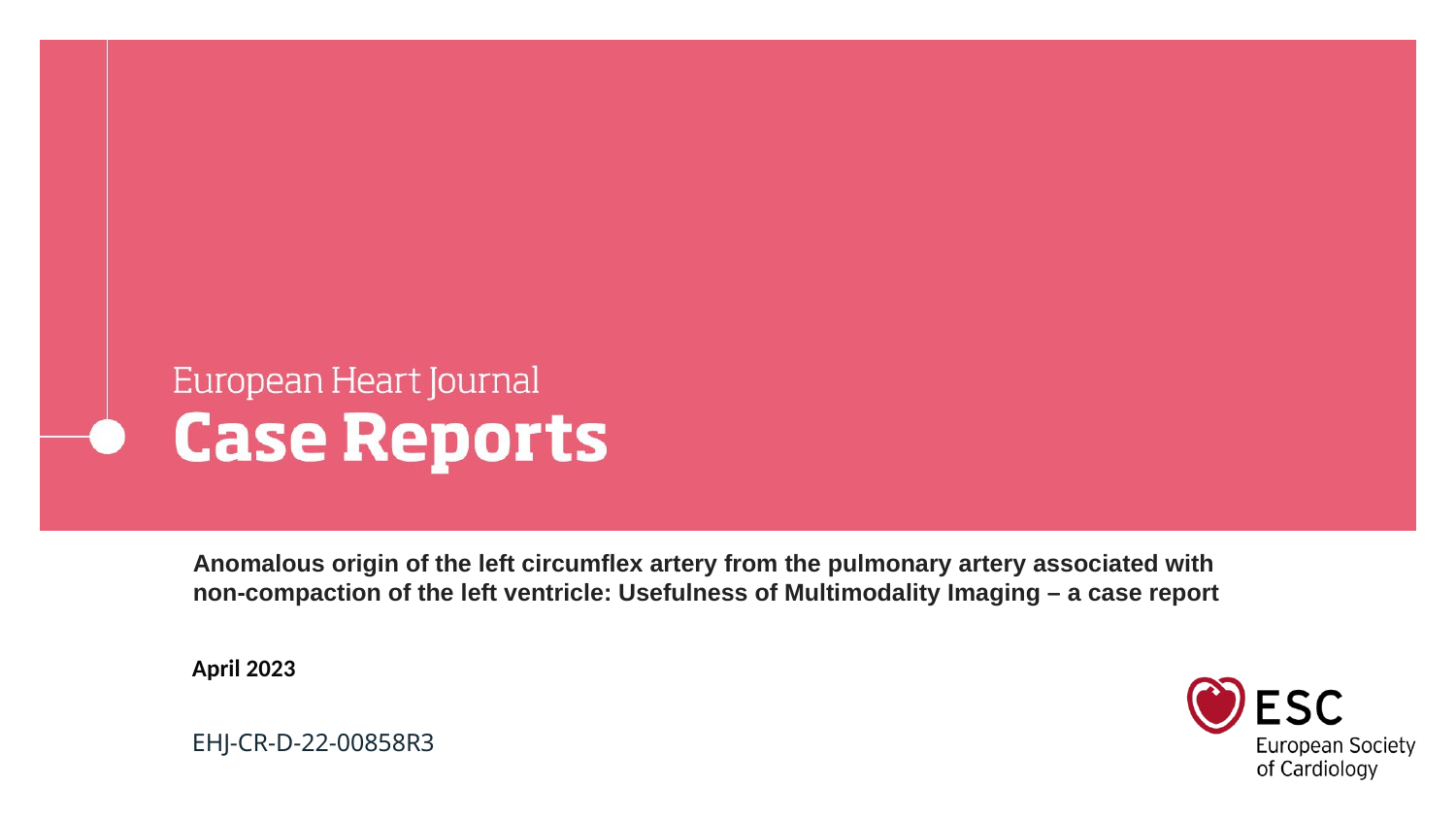

# Anomalous origin of the left circumflex artery from the pulmonary artery associated with non-compaction of the left ventricle: Usefulness of Multimodality Imaging – a case report
April 2023
EHJ-CR-D-22-00858R3

## Slide 2
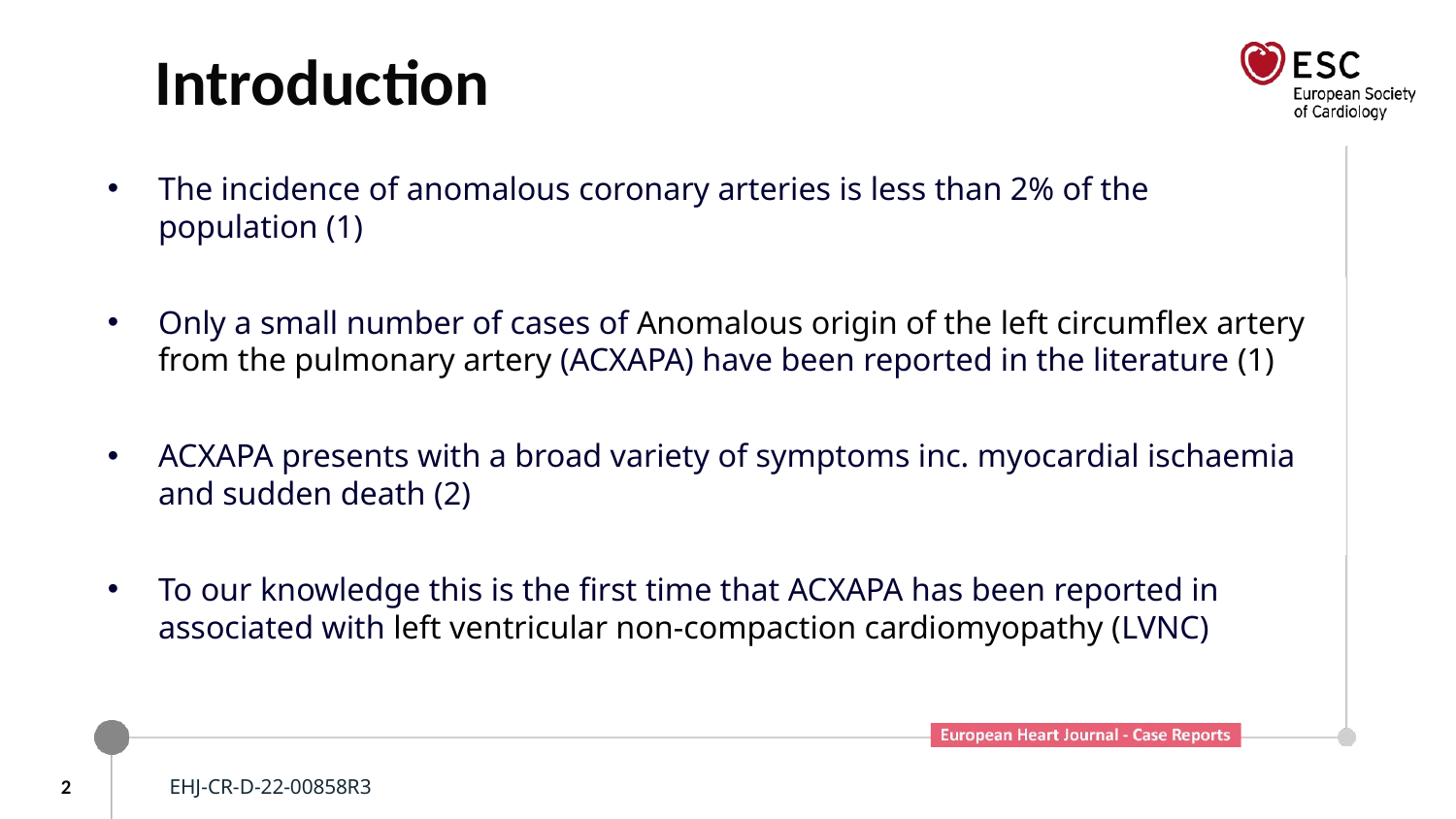

# Introduction
The incidence of anomalous coronary arteries is less than 2% of the population (1)
Only a small number of cases of Anomalous origin of the left circumflex artery from the pulmonary artery (ACXAPA) have been reported in the literature (1)
ACXAPA presents with a broad variety of symptoms inc. myocardial ischaemia and sudden death (2)
To our knowledge this is the first time that ACXAPA has been reported in associated with left ventricular non-compaction cardiomyopathy (LVNC)
2
EHJ-CR-D-22-00858R3

## Slide 3
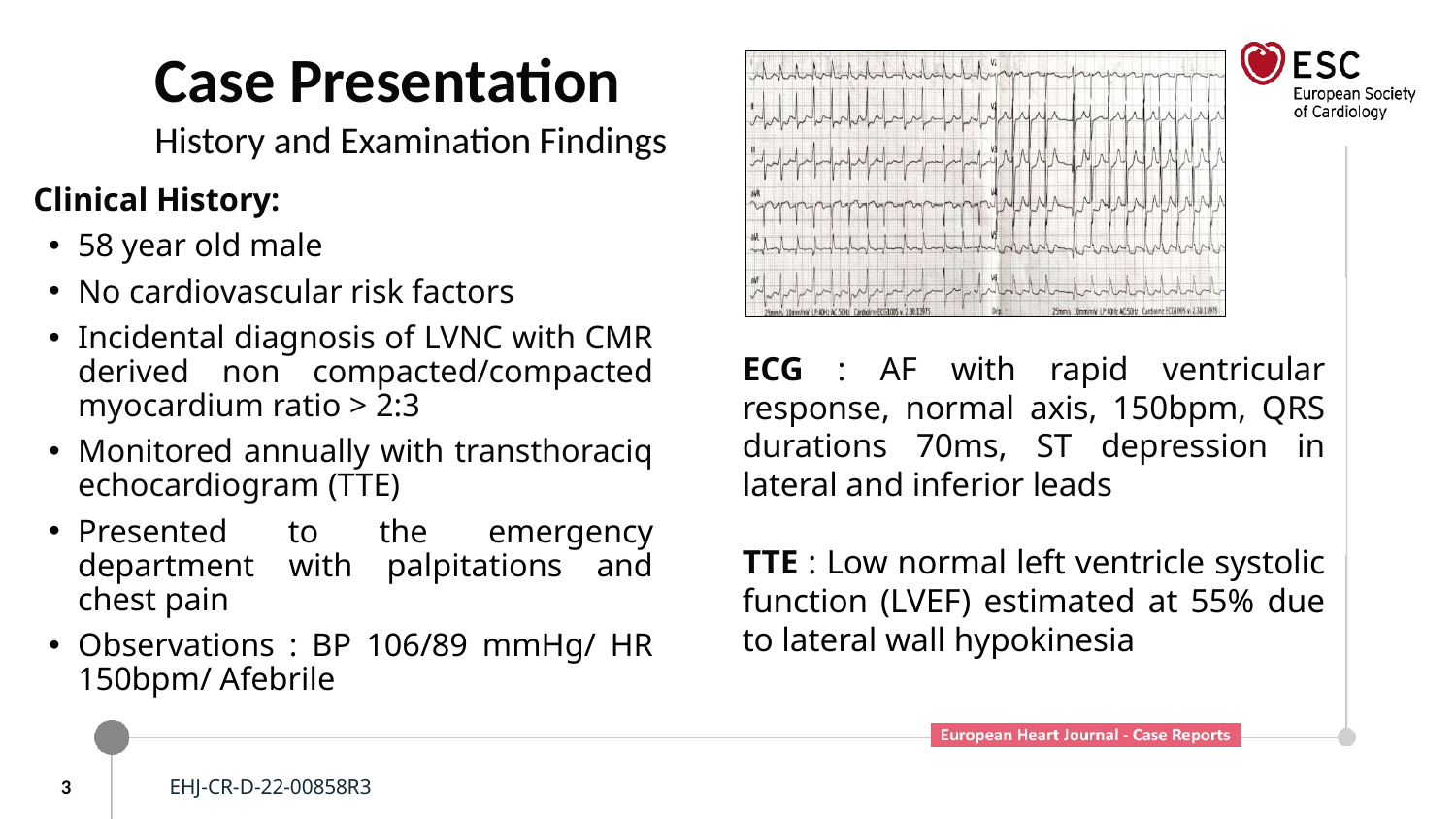

# Case Presentation History and Examination Findings
Clinical History:
58 year old male
No cardiovascular risk factors
Incidental diagnosis of LVNC with CMR derived non compacted/compacted myocardium ratio > 2:3
Monitored annually with transthoraciq echocardiogram (TTE)
Presented to the emergency department with palpitations and chest pain
Observations : BP 106/89 mmHg/ HR 150bpm/ Afebrile
ECG : AF with rapid ventricular response, normal axis, 150bpm, QRS durations 70ms, ST depression in lateral and inferior leads
TTE : Low normal left ventricle systolic function (LVEF) estimated at 55% due to lateral wall hypokinesia
3
EHJ-CR-D-22-00858R3

## Slide 4
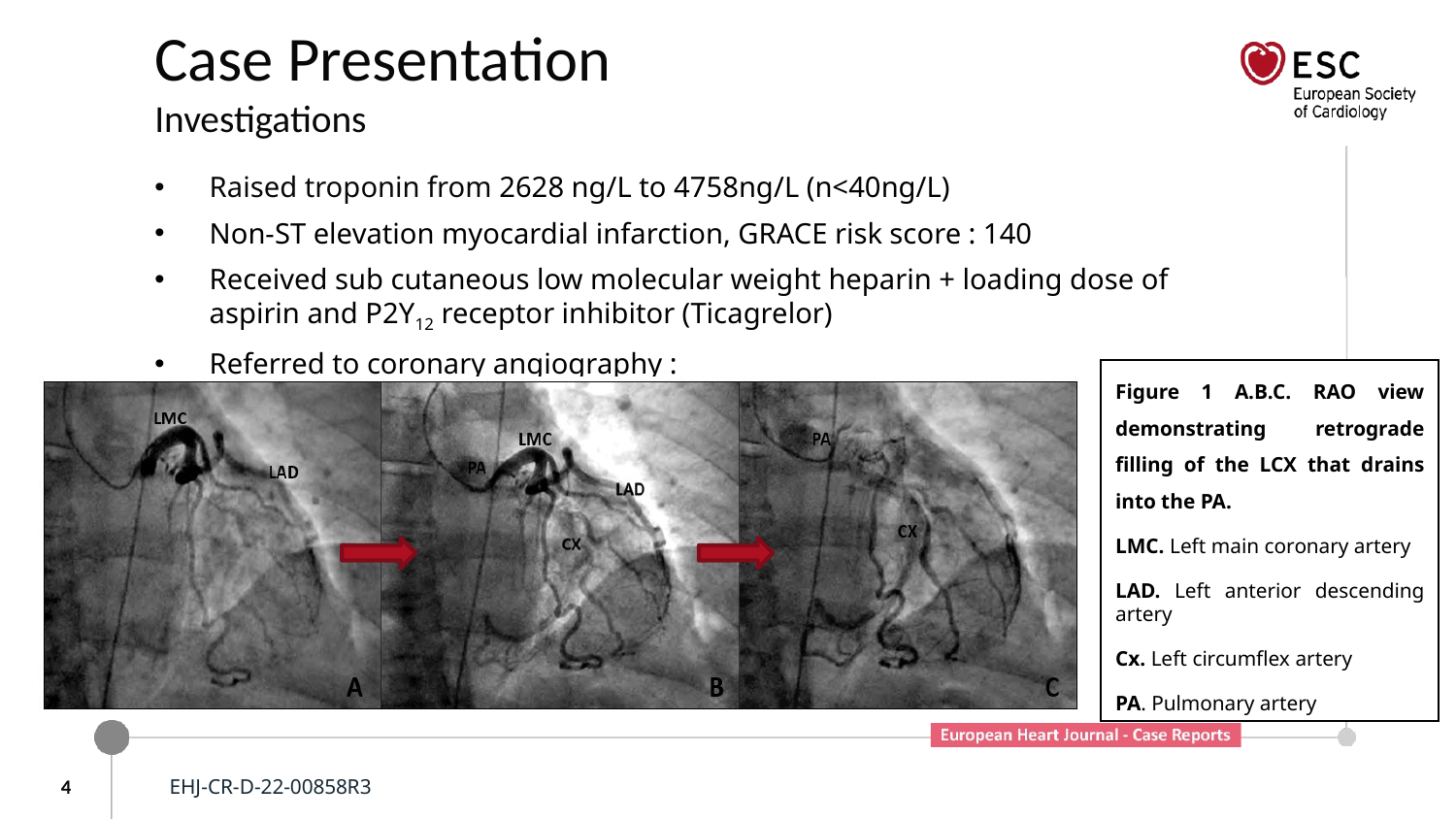

# Case Presentation Investigations
Raised troponin from 2628 ng/L to 4758ng/L (n<40ng/L)
Non-ST elevation myocardial infarction, GRACE risk score : 140
Received sub cutaneous low molecular weight heparin + loading dose of aspirin and P2Y12 receptor inhibitor (Ticagrelor)
Referred to coronary angiography :
Figure 1 A.B.C. RAO view demonstrating retrograde filling of the LCX that drains into the PA.
LMC. Left main coronary artery
LAD. Left anterior descending artery
Cx. Left circumflex artery
PA. Pulmonary artery
4
EHJ-CR-D-22-00858R3

## Slide 5
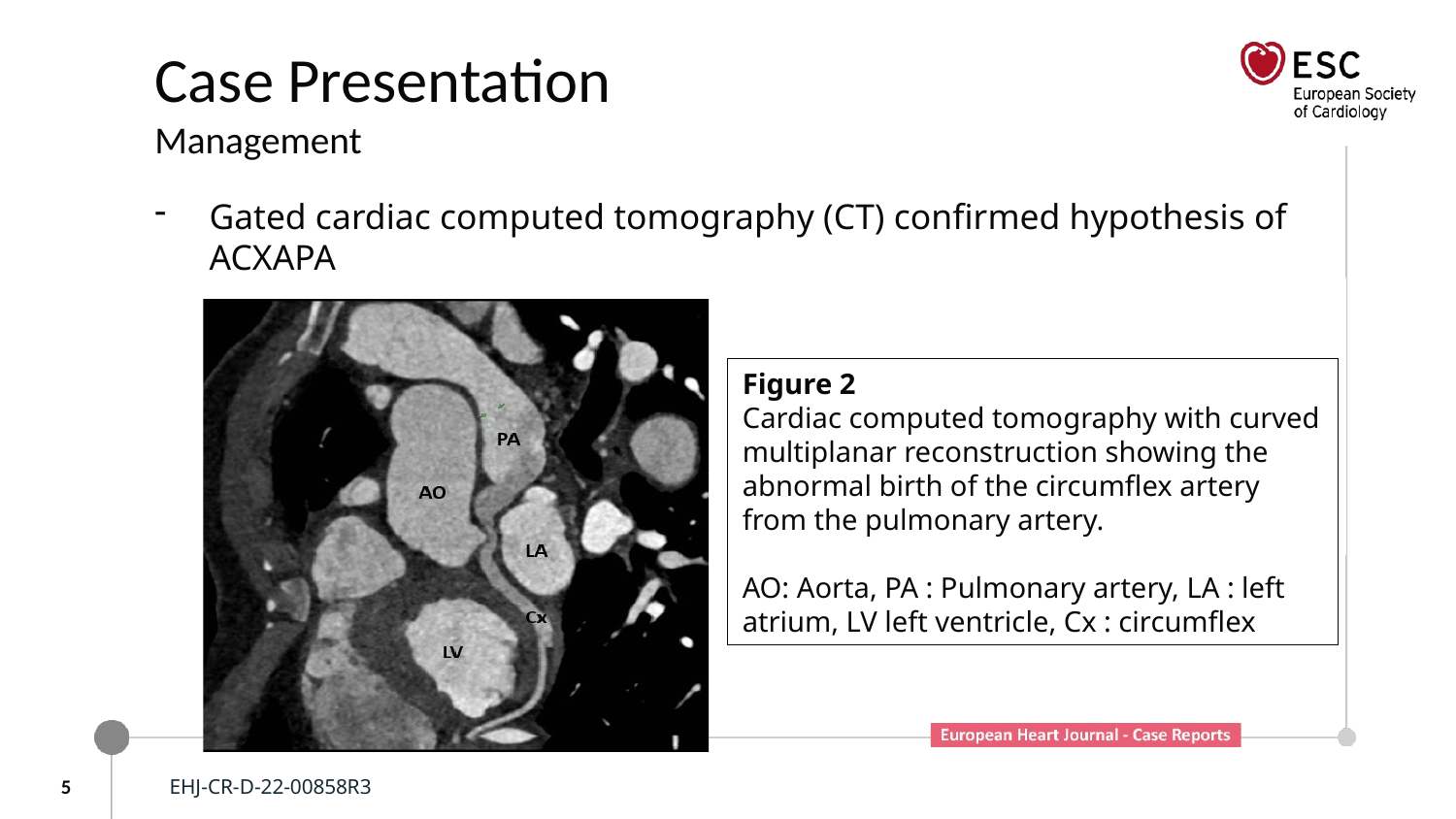

# Case Presentation Management
Gated cardiac computed tomography (CT) confirmed hypothesis of ACXAPA
Figure 2
Cardiac computed tomography with curved multiplanar reconstruction showing the abnormal birth of the circumflex artery from the pulmonary artery.
AO: Aorta, PA : Pulmonary artery, LA : left atrium, LV left ventricle, Cx : circumflex
5
EHJ-CR-D-22-00858R3

## Slide 6
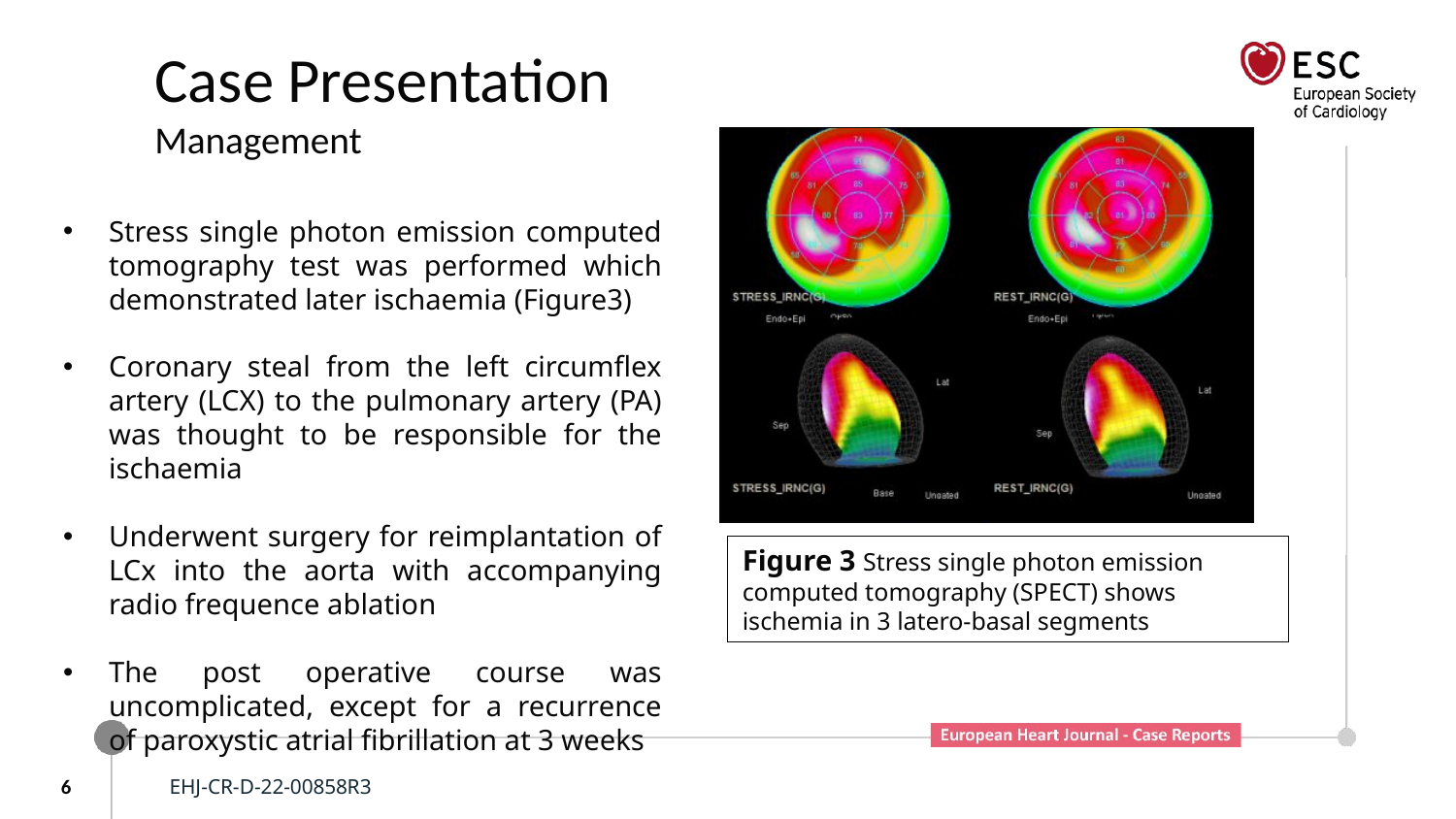

# Case Presentation Management
Stress single photon emission computed tomography test was performed which demonstrated later ischaemia (Figure3)
Coronary steal from the left circumflex artery (LCX) to the pulmonary artery (PA) was thought to be responsible for the ischaemia
Underwent surgery for reimplantation of LCx into the aorta with accompanying radio frequence ablation
The post operative course was uncomplicated, except for a recurrence of paroxystic atrial fibrillation at 3 weeks
Figure 3 Stress single photon emission computed tomography (SPECT) shows ischemia in 3 latero-basal segments
6
EHJ-CR-D-22-00858R3

## Slide 7
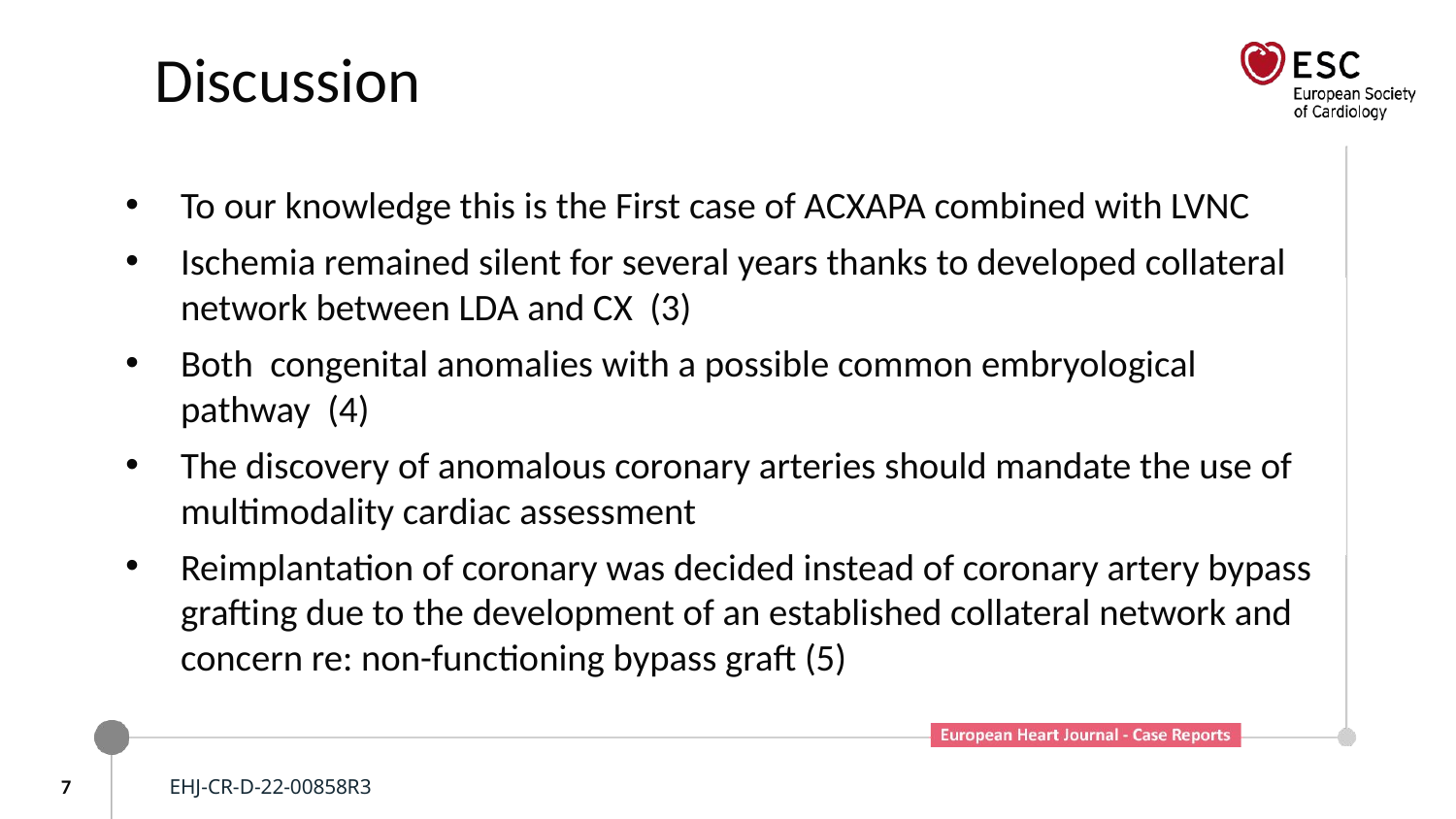

# Discussion
To our knowledge this is the First case of ACXAPA combined with LVNC
Ischemia remained silent for several years thanks to developed collateral network between LDA and CX (3)
Both congenital anomalies with a possible common embryological pathway (4)
The discovery of anomalous coronary arteries should mandate the use of multimodality cardiac assessment
Reimplantation of coronary was decided instead of coronary artery bypass grafting due to the development of an established collateral network and concern re: non-functioning bypass graft (5)
7
EHJ-CR-D-22-00858R3

## Slide 8
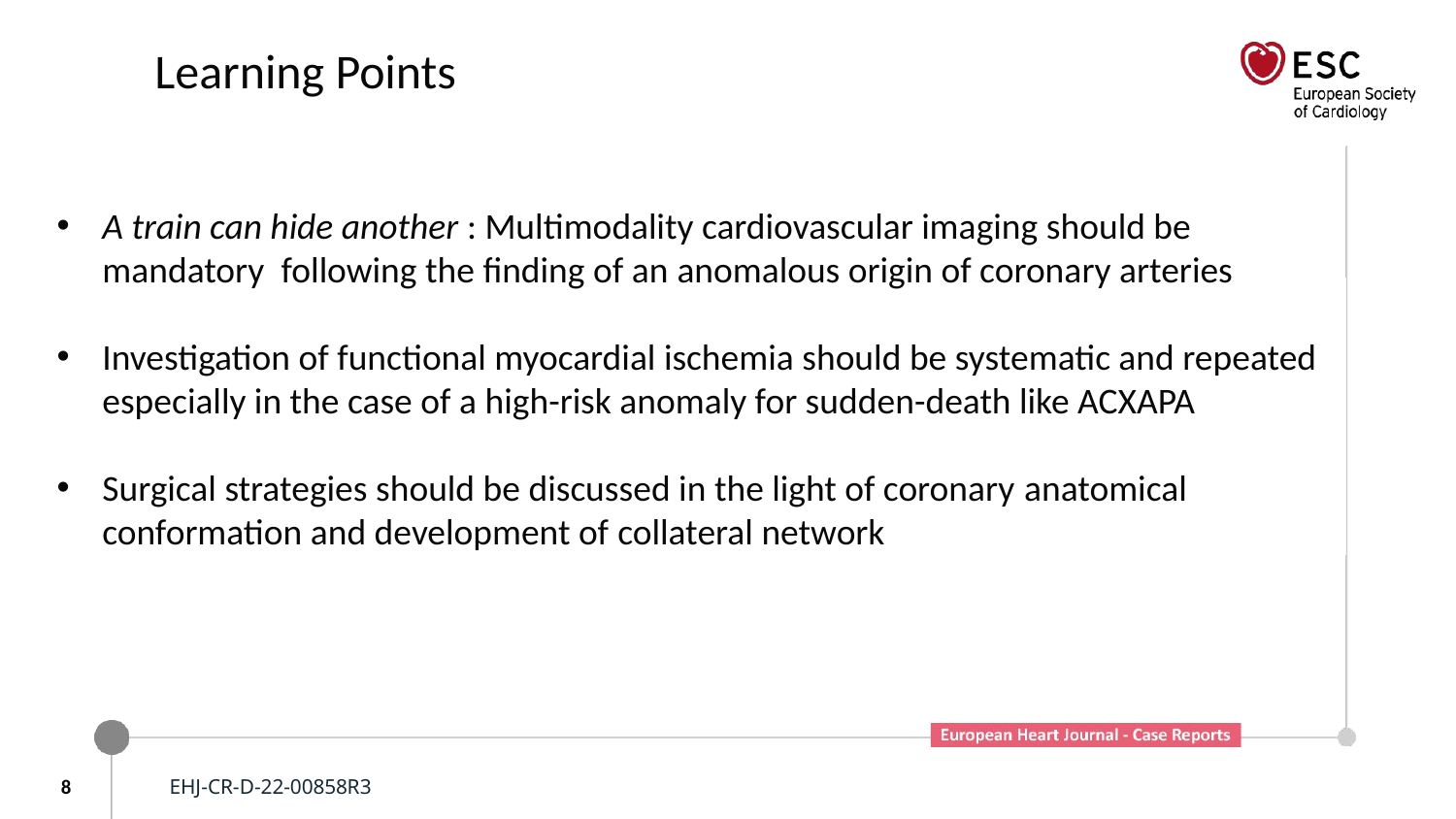

# Learning Points
A train can hide another : Multimodality cardiovascular imaging should be mandatory following the finding of an anomalous origin of coronary arteries
Investigation of functional myocardial ischemia should be systematic and repeated especially in the case of a high-risk anomaly for sudden-death like ACXAPA
Surgical strategies should be discussed in the light of coronary anatomical conformation and development of collateral network
8
EHJ-CR-D-22-00858R3

## Slide 9
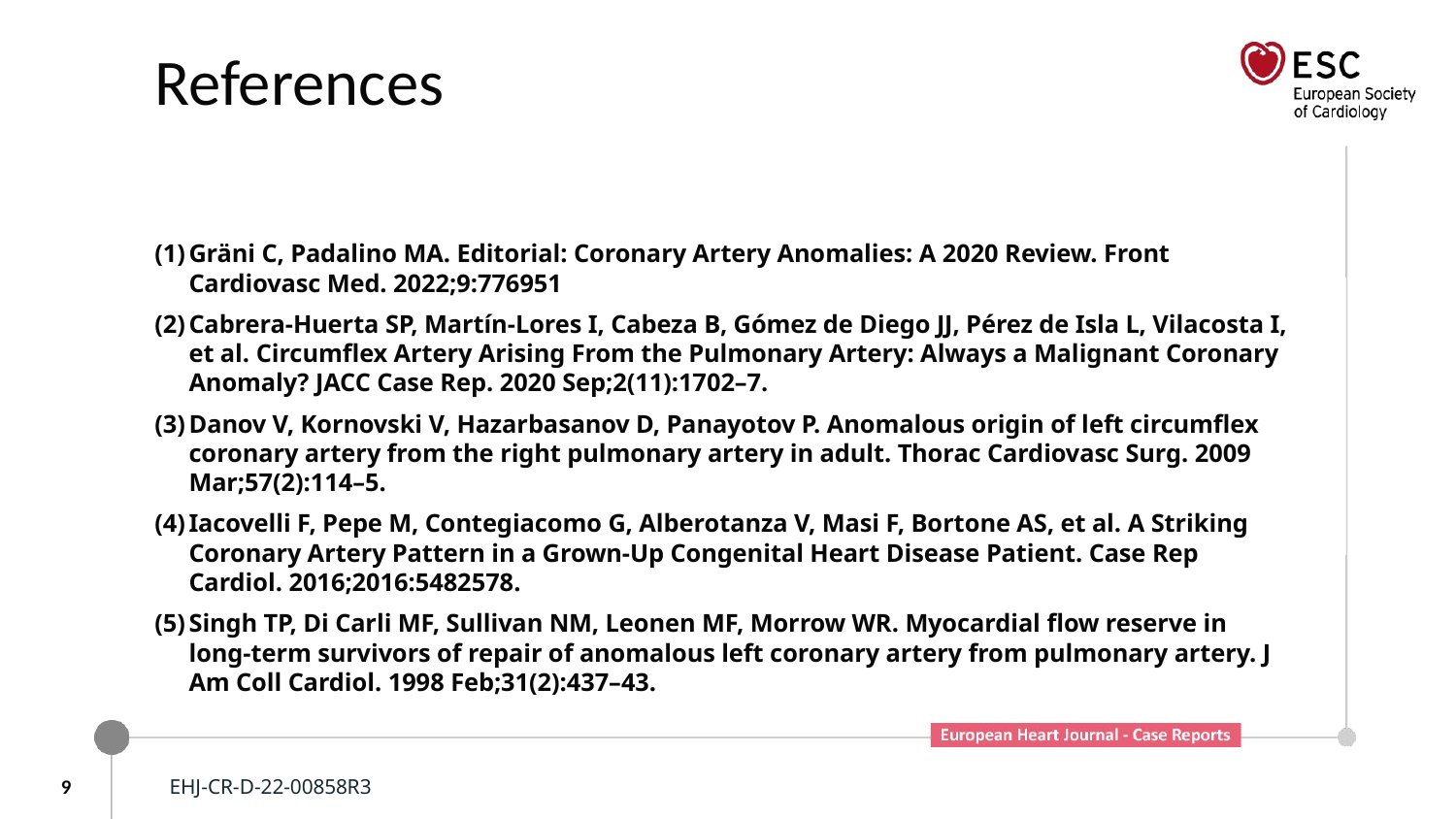

# References
Gräni C, Padalino MA. Editorial: Coronary Artery Anomalies: A 2020 Review. Front Cardiovasc Med. 2022;9:776951
Cabrera-Huerta SP, Martín-Lores I, Cabeza B, Gómez de Diego JJ, Pérez de Isla L, Vilacosta I, et al. Circumflex Artery Arising From the Pulmonary Artery: Always a Malignant Coronary Anomaly? JACC Case Rep. 2020 Sep;2(11):1702–7.
Danov V, Kornovski V, Hazarbasanov D, Panayotov P. Anomalous origin of left circumflex coronary artery from the right pulmonary artery in adult. Thorac Cardiovasc Surg. 2009 Mar;57(2):114–5.
Iacovelli F, Pepe M, Contegiacomo G, Alberotanza V, Masi F, Bortone AS, et al. A Striking Coronary Artery Pattern in a Grown-Up Congenital Heart Disease Patient. Case Rep Cardiol. 2016;2016:5482578.
Singh TP, Di Carli MF, Sullivan NM, Leonen MF, Morrow WR. Myocardial flow reserve in long-term survivors of repair of anomalous left coronary artery from pulmonary artery. J Am Coll Cardiol. 1998 Feb;31(2):437–43.
9
EHJ-CR-D-22-00858R3
